# Complexity, centralization, and fragility in economic networks

**DOI:** 10.1371/journal.pone.0208265

**Published:** 2018-11-29

**Authors:** Carlo Piccardi, Lucia Tajoli

**Affiliations:** 1 Department of Electronics, Information, and Bioengineering, Politecnico di Milano, Milano, Italy; 2 Department of Management, Economics, and Industrial Engineering, Politecnico di Milano, Milano, Italy; Uniiversity of Padova, ITALY

## Abstract

Trade networks, across which countries distribute their products, are crucial components of the globalized world economy. Their structure affects the mechanism of propagation of shocks from country to country, as observed in a very sharp way in the past decade, characterized by economic uncertainty in many parts of the world. Such trade structures are strongly heterogeneous across products, given the different features of the countries which buy and sell goods. By using a diversified pool of indicators from network science and product complexity theory, we quantitatively demonstrate that, overall, products with higher complexity—i.e., with larger technological content and/or number of components—are traded through more centralized networks—i.e., with a smaller number of countries concentrating most of the export flow. Since centralized networks are known to be more vulnerable, we argue that the current composition of production and trading is associated to high fragility at the level of the most complex—thus strategic—products.

## Introduction

How fragile is the world economy? Given the increasing globalization of economic systems, will economic shocks have widespread diffusion to all countries? The recent evidence of the international financial crisis of 2007-2008 and the European debt crisis suggest that indeed most of the world countries are highly exposed. According to the International Monetary Fund [1], very few countries were spared the trade slowdown that followed the crisis. Also, non-economic events hitting a specific economy have a broad impact: the volcano eruption in Iceland in 2010 and the earthquake in Japan in 2011 generated significant effects on production, not only in the countries directly hit, but also in a number of other linked economies [2, 3]. These events stirred a debate on the relevance and persistence of transmission of such economic shocks, which however has not reached a definitive conclusion. On the one hand, the growing globalization and the increased diversification of economic links between countries [4, 5] give support to the idea that in this context, because of diversification, specific shocks should average out, and produce negligible general effects [6], making countries more resilient. If a country exports to a diversified group of countries, each with an independent business cycle, the probability that a shock hitting one of its destination markets significantly affects its export is small (for country *i*, it is proportional to the share of export to the country *j* subject to shock over the total export of *i* to the world [7]). Furthermore, a country specific shock can be counterbalanced by a shock of opposite sign elsewhere, with a net effect close to zero. On the other hand, the presence of many interconnections between countries is a potential strong propagation mechanism increasing the total exposure of each country, and the high density of the world trade network helps the rapid diffusion of shocks [8, 9]. Which of the two effects prevails depends on the specific structure of these interconnections. In an economic system, the diversification argument to reduce the impact of shocks applies in a symmetric structure of links [6], but it needs not to be valid if strong asymmetries occur. This is why the analysis of the centralization of the global trading system is important.

This work contributes to this debate by showing that the high density of the economic links among countries occurs together with a very uneven distribution of such links. Using different indicators, we consistently observe that the world trade network is highly centralized in many industries, and notably we show that complex and high-tech goods typically display a stronger centralization of their trade structure. A fundamental result in network science is that the transmission of shocks—and therefore the vulnerability of the system—is related to structure, with highly centralized networks being the most fragile [10–13]: in such networks, failures or perturbations originating in the most central nodes rapidly propagates through the network, heavily compromising the function of the entire system. This feature has also been thoroughly discussed in the context of international trade, where models have been proposed capable of describing the contrasting ways of shock propagation in networks with different degrees of centralization (e.g., [6, 14–16]). The hypothesis of independent business cycles and idiosyncratic shocks does not hold in a centralized world system with strong, non-random and asymmetric trade links. In such a context, through trade, changes in demand and production at the country level propagate from one node to another in a non-random fashion and need not to average out, as discussed also by [17]. Given that complex goods are very relevant for all economies, and that high-tech industries—according to World Bank estimates—make up about one fifth of all world trade, we argue that the current composition of production is potentially associated to high fragility of the trading system, making it vulnerable to attacks or disasters. The impact of shocks hitting the central nodes in these industries can be large and widespread.

## Methods

We analyze data of inter-country trade in year 2014 among 223 countries, extracted from the CEPII-BACI database [18] with HS 4-digit classification, wich defines 1,242 products. We denote by *E* = [*e*_*cp*_] the 223×1,242 country/product trade matrix, whose entry *e*_*cp*_ is the export value (in USD) of product *p* by country *c*, and by *M* = [*m*_*cp*_] the binarized country/product matrix, whose entry *m*_*cp*_ is 1 if the *revealed comparative advantage* *r*_*cp*_ that country *c* has in product *p* is greater or equal than 1, and 0 otherwise. We recall that

$$\begin{array}{c} r_{cp}=\frac{e_{cp}}{\sum_{p^{\prime }}e_{cp^{\prime }}}/\frac{\sum_{c^{\prime }}e_{c^{\prime }p}}{\sum_{c^{\prime }p^{\prime }}e_{c^{\prime }p^{\prime }}}. \end{array}$$ (1)

### Measuring product complexity

The role of products’ complexity to understand trade patterns among countries has been highlighted only recently in the economics literature [19, 20], and there is not a general consensus on the definition of complex goods. Therefore, several proposals have been put forward to quantify the complexity of a product. To robustify our analysis, we consider three different indicators whose values are computed (or are publicly available) for each one of the products *p*.

#### Hidalgo-Hausmann (HH) index $X_{p}^{\prime }$

It is the *Product Complexity Index* defined in [21, 22], which provides a ranking of *“the amount and sophistication of know-how required to produce a product”* (see http://atlas.cid.harvard.edu/learn/glossary/). Consider the 1,242×1,242 square matrix $\tilde{M}=\left[{\tilde{m}}_{pp^{\prime }}\right]$ defined by

$$\begin{array}{c} {\tilde{m}}_{pp^{\prime }}=\sum_{c}\frac{m_{cp}m_{cp^{\prime }}}{k_{c,0}k_{p,0}}, \end{array}$$ (2)

where *k*_*c*,0_ = ∑_*p*_ *m*_*cp*_ and *k*_*p*,0_ = ∑_*c*_ *m*_*cp*_. Then $X_{p}^{\prime }$ is the *p*-th entry of the eigenvector of $\tilde{M}$ associated with the second largest eigenvalue, after the entries of the eigenvector have been normalized by their mean and standard deviation [22]. We downloaded the values of $X_{p}^{\prime }$ for the year of interest (2014) from the website of The Atlas of Economic Complexity (http://atlas.cid.harvard.edu/rankings/product/2014/).

We remark that, after its first proposal [21], the interpretation and the mathematical properties of the product complexity index (and of its companion index for ranking countries) have been thoroughly discussed and criticized. This debate is however out of the scope of this paper—the reader is referred e.g. to [23–26].

#### Fitness-Complexity (FC) index $X_{p}^{\prime \prime }$

It is the product complexity measure proposed in [24] (extensive metrics form), obtained elaborating on the above HH approach and based on the following non-linear iterative computation:

$$\begin{array}{ccc} & {\tilde{Q}}_{p}^{\left(n\right)}=\frac{1}{\sum_{c}q_{cp}/F_{c}^{\left(n-1\right)}}, & {\tilde{F}}_{c}^{\left(n\right)}=\sum_{p}q_{cp}Q_{p}^{\left(n-1\right)}, \end{array}$$ (3)

$$\begin{array}{ccc} & Q_{p}^{\left(n\right)}={\tilde{Q}}_{p}^{\left(n\right)}/{\left\langle {\tilde{Q}}_{p}^{\left(n\right)}\right\rangle }_{p}, & F_{c}^{\left(n\right)}={\tilde{F}}_{c}^{\left(n\right)}/{\left\langle {\tilde{F}}_{c}^{\left(n\right)}\right\rangle }_{c}, \end{array}$$ (4)

where *q*_*cp*_ = *e*_*cp*_/∑_*c*′_ *e*_*c*′*p*_ and $Q_{p}^{\left(0\right)}=1$ ∀*p*, $F_{c}^{\left(0\right)}=1$ ∀*c*. While [24] empirically reported the convergence of the above iterations to bounded non-zero values, recent studies discussed that, with specific structures of the country/product trade matrix, a number of entries of the fitness/complexity vectors $F_{c}^{\left(n\right)},Q_{p}^{\left(n\right)}$ may tend to zero as *n* increases [27, 28]. In our work, we used the termination criterion proposed in [27] (Eq. (E.1)): convergence to non-zero values is reached for all countries/products after 254 iterations. Eventually, we take the product complexity $X_{p}^{\prime \prime }$ as the logarithm of the limit value of $Q_{p}^{\left(n\right)}$.

#### PRODY index $X_{p}^{\prime \prime \prime }$

It is the (weighted) average income per-capita of the countries exporting product *p* [29]:

$$\begin{array}{c} X_{p}^{\prime \prime \prime }=\sum_{c}\frac{s_{cp}}{\sum_{c^{\prime }}s_{c^{\prime }p}}I_{c}, \end{array}$$ (5)

where *s*_*cp*_ = *e*_*cp*_/∑_*p*_ *e*_*cp*_ is the share of product *p* in the export basket of country *c*, and *I*_*c*_ is the income of country *c* measured as GDP per capita adjusted for power purchasing parity (data source: The World Bank, https://data.worldbank.org/).

Not surprisingly, the three above indicators are overall positively correlated (see Fig A in S1 File), yet they display remarkable differences on many products, as discussed in Refs. [21, 24]. In fact, the HH and the FC complexity indexes more explicitly take into account countries’ characteristics and capabilities enabling the export of complex products, while the PRODY indicator is a more indirect—and possibly less contingent on trade—measure, being based on income per capita.

### Measuring network centralization

**Fig 1:**
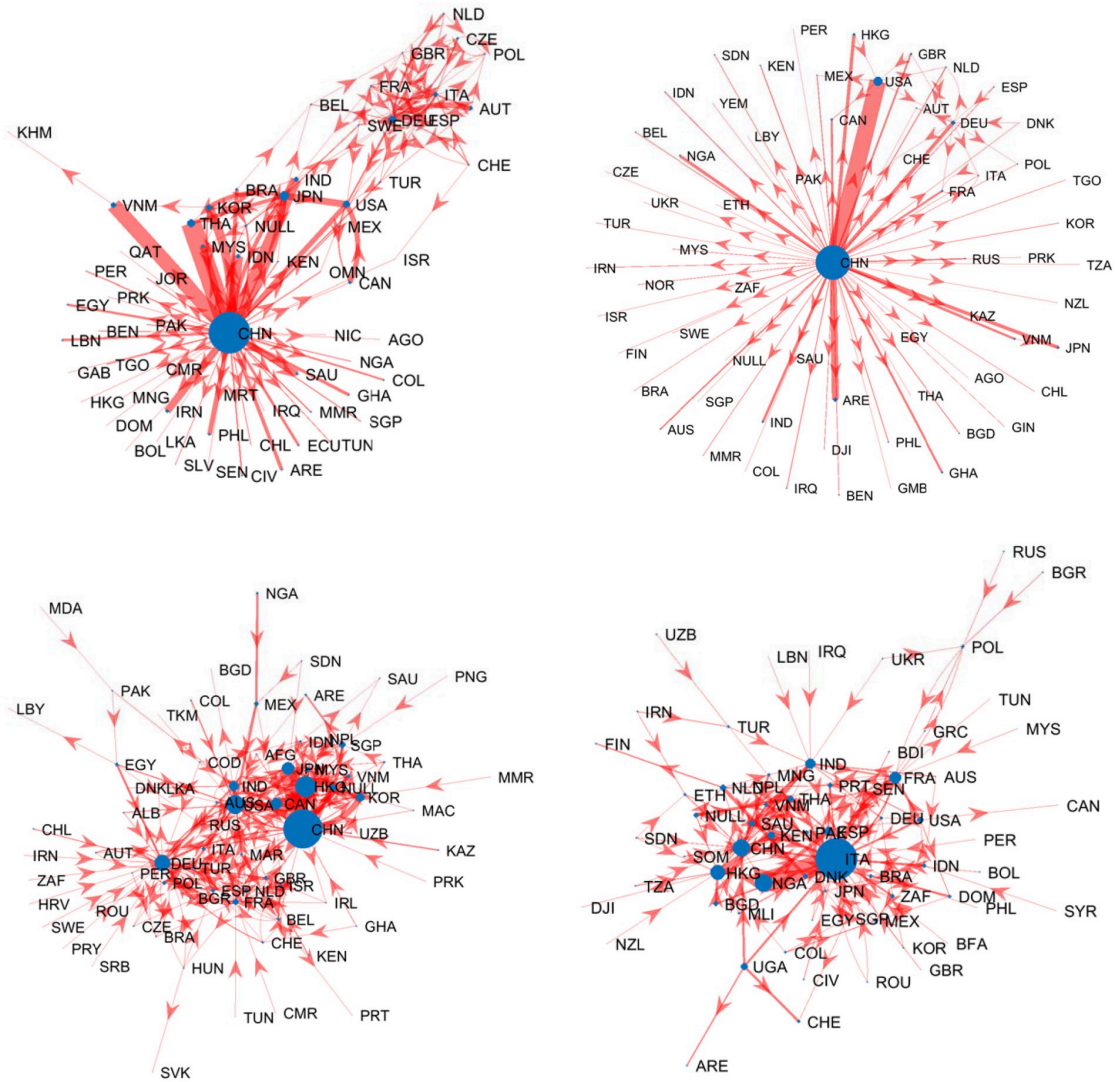
Four examples of product trade networks. Products #7227 (top-left, “Bars and rods, hot-rolled, in irregularly wound coils, of other alloy steel”, Section “Metals”) and #8513 (top-right, “Portable electric lamps designed to function by their own source of energy”, Section “Machinery/Electrical”) display strong centralization, with most of the export concentrated in one or a few countries and a predominant star-like topology. Products #1211 (bottom-left, “Plants and parts of plants, including seeds and fruits”, Section “Vegetable Products”) #4106 (bottom-right, “Tanned or crust hides and skins of other animals, without wool or hair on”, Section “Raw Hides, Skins, Leather & Furs”), on the contrary, are characterized by a few leading countries relating to many others by an intricate pattern of connections. To improve visualization, links carrying less than 1% of the largest weight have not been displayed.

For each product *p*, trade data define a weighted, directed network *N*_*p*_ where the weight $w_{ij}^{p}$ of the link from country *i* to country *j* is the monetary value of the export from *i* to *j* (Fig 1). For each product, we analyze the *largest weakly connected component* (e.g., Ref. [30]) of the trade network, so preserving directionality and weights but removing isolated nodes (i.e., countries not participating in the trade of that product) or small isolated subnetworks (see Fig B in S1 File).

In general terms, a centralization index aims at capturing to what extent a given property is unevenly distributed among network nodes. We are interested, for each product, in quantifying the heterogeneity in the export capabilities of countries. We quantify centralization by three different indicators which not only describe local features (country exports) but also dynamical and robustness properties dependent on the global network structure. All the three indicators are normalized to take values in the [0, 1] range, with zero (resp. one) denoting minimal (resp. maximal) centralization.

Before defining centralization indices in detail, we mention that a structural measure that can be used in alternative to centralization is *nestedness*. In the context of world trade, this approach has been applied to the bipartite country/product trade matrix [31, 32] or, more recently, to a multilayer representation of the World Input-Output Dataset [33]. Our work adopts a different line of investigation, in a twofold manner: we compute a structural measure of hetereogeneity (i.e., centralization, in our case) for each single product network, rather than for the entire (bipartite or multilayer) network; and we discuss the relationship of such a measure with product complexity, a point untouched in the above quoted contributions.

#### GINI index $Y_{p}^{\prime }$

**Fig 2:**
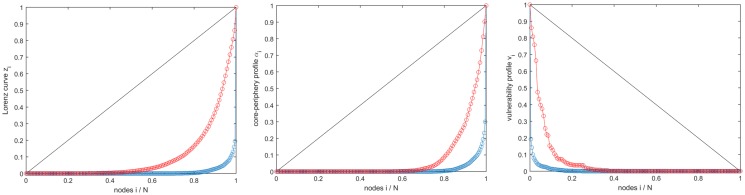
Computation of the centralization indexes. Left panel: Lorenz curve (GINI index); Central panel: core-periphery profile (CP index); Right panel: vulnerability profile (VI index). The value of the centralization indexes is the (normalized) area between the curve and the diagonal line. In each panel, the blue curve corresponds to a product with higher centralization (product #8513), the red one to a product with smaller centralization (product #4106): the trade networks for these two products are in Fig 1.

A standard Gini index can straightforwardly be used to quantify the unevenness of the distribution of the out-strengths (i.e., the country total export of product *p*). Let *W* = [*w*_*ij*_] be the weight matrix (we omit the product index *p* to keep the notation simpler), $s_{i}^{out}=\sum_{j}w_{ij}$ the out-strength of node *i*, and $\tilde{W}=\left[{\tilde{w}}_{ij}\right]$ the weight matrix after nodes have been re-ordered by increasing out-strength, i.e., ${\tilde{s}}_{1}^{out}\leq {\tilde{s}}_{2}^{out}\leq \ldots \leq {\tilde{s}}_{n}^{out}$, where *n* is the number of nodes. We define the *Lorenz (cumulated) curve* as *z*_0_ = 0, $z_{i}=\left(1/S\right)\sum_{j=1}^{i}{\tilde{s}}_{j}^{out}$, *i* = 1, 2, …, *n*, where *S* = ∑_*ij*_ *w*_*ij*_ is the total trade. In the case of least centralization, i.e., all nodes having the same out-strength ($s_{i}^{out}=S/n$ ∀*i*), the sequence *z*_*i*_ is linearly increasing from 0 to 1. In the opposite case, i.e., only one node has nonzero export while all the others are pure importers, we have *z*_0_ = *z*_1_ = … = *z*_*n*−1_ = 0, *z*_*n*_ = 1. The Gini index $Y_{p}^{\prime }$ is the normalized distance of the cumulated curve from the linearly increasing curve, as shown in Fig 2, so that $Y_{p}^{\prime }=0$ for the network with homogeneous out-strength, and $Y_{p}^{\prime }=1$ for the star network with the center as the only exporter.

#### Core-Periphery (CP) index $Y_{p}^{\prime \prime }$

It is the *core-periphery score* defined in Ref. [34] and based on the notion of *persistence probability* *α*_*S*_ of a subnetwork *S*, namely the probability that a random walker currently in any of the nodes of *S* remains in *S* at the next time step [34, 35]. A network profile is built by ordering nodes from the periphery to the core according to a heuristic strategy. We start by the node *i* with minimal strength, and we generate a sequence *S*_1_ ⊆ *S*_2_ ⊆ … ⊆ *S*_*n*_ of subnetworks, where *S*_1_ = {*i*} is the initial node and *S*_*n*_ = {1, 2, …, *n*} is the whole network, by adding at each step *k* the node attaining the minimal persistence probability *α*_*k*_ of the subnetwork *S*_*k*_. The obtained sequence 0 = *α*_1_ ≤ *α*_2_ ≤ … ≤ *α*_*n*_ = 1 is the *core-periphery profile* of the network. The complete network and the star network are extreme cases for the core-periphery profile. The former has no core-periphery structure as all nodes are equivalent, so that *α*_*k*_ = (*k* − 1)/(*n* − 1) grows linearly from 0 to 1, while the latter is the most centralized network and has *α*_1_ = *α*_2_ = … = *α*_*n*−1_ = 0, *α*_*n*_ = 1. The centralization $Y_{p}^{\prime \prime }$ is defined as the normalized distance of the core-periphery profile from that of the complete network (Fig 2), so that $Y_{p}^{\prime \prime }=0$ for the complete network and $Y_{p}^{\prime \prime }=1$ for the star network.

#### Vulnerability index (VI) $Y_{p}^{\prime \prime \prime }$

It is based on Ref. [36] and measures how rapidly the aggregated network weight is lost when connectivity decreases because nodes are subsequently removed starting from those with largest out-strength. We re-order nodes by decreasing out-strength, thus ${\tilde{s}}_{1}^{out}\geq {\tilde{s}}_{2}^{out}\geq \ldots \geq {\tilde{s}}_{n}^{out}$, and we define the *vulnerability profile* as 1 = *v*_0_ ≥ *v*_1_ ≥ … ≥ *v*_*n*_ = 0, where *v*_*k*_ is the total weight of the network after nodes {1, 2, …, *k*} have been removed, divided by the total weight *S* of the original network. The vulnerability profile falls immediately to zero for a star network (*v*_1_ = 0) whereas it decays linearly for a complete network with homogeneous weights. We take as $Y_{p}^{\prime \prime \prime }$ the normalized distance of the profile from that of the complete network (Fig 2), so that $Y_{p}^{\prime \prime \prime }=0$ for the complete network and $Y_{p}^{\prime \prime \prime }=1$ for the star network.

## Results

**Fig 3:**
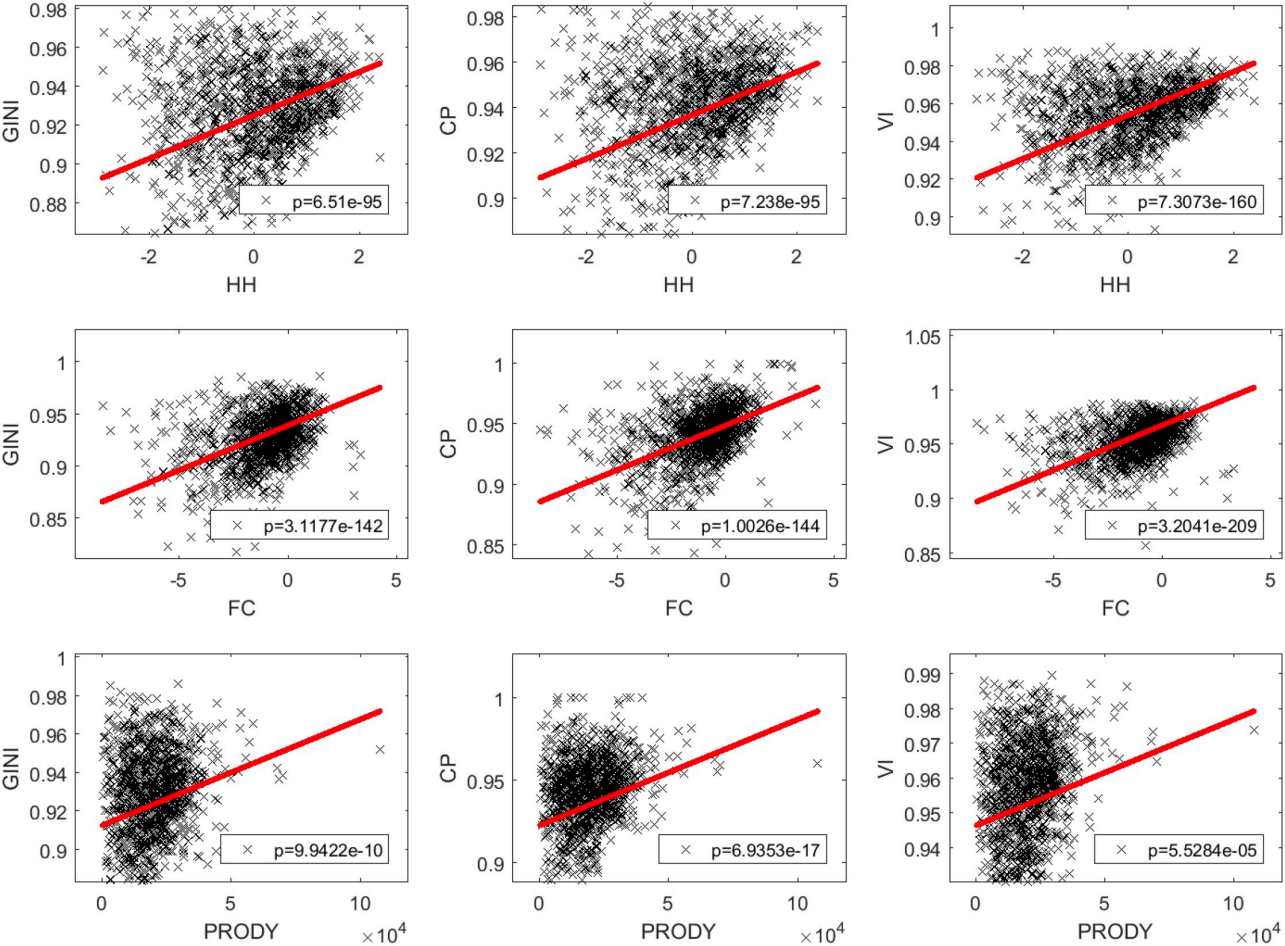
Complexity vs centralization in export data. For the 3 × 3 combinations of indicators, the scatter plots report the complexity/centralization values for the complete set of 1,242 products. The weighted linear regression consistently displays statistically significant positive slope (see p-value in the bottom-right corner). In each plot, the regression line (red) is drawn in the range spanned by the complexity index on the abscissa.

For each one of the 3 × 3 complexity/centralization pairs (*X*_*p*_, *Y*_*p*_) we obtain a scatter plot with one point for each of the 1,242 products *p* (Fig 3). We conjecture that the more complex is a product, the more centralized is its distribution network *N*_*p*_, given that countries with the necessary skills and organization capacity to produce complex products are few compared to countries able to produce and export efficiently simple products. The international trade literature shows indeed that complex goods are more difficult to export, and doing so requires a country to be endowed with the appropriate institutions [37]. Therefore, we expect that the selection of this type of exporters can give rise to more centralized networks. To test this conjecture, we compute the least-squares linear interpolant for each scatter plot. Since the economic importance of products is largely different (see Fig B in S1 File), we compute a weighted regression, the weight for point (*X*_*p*_, *Y*_*p*_) being the total world export $\sum_{ij}w_{ij}^{p}$ of product *p*. We expect a positive slope of the linear interpolant, and we check the statistical significance of the result.

**Fig 4:**
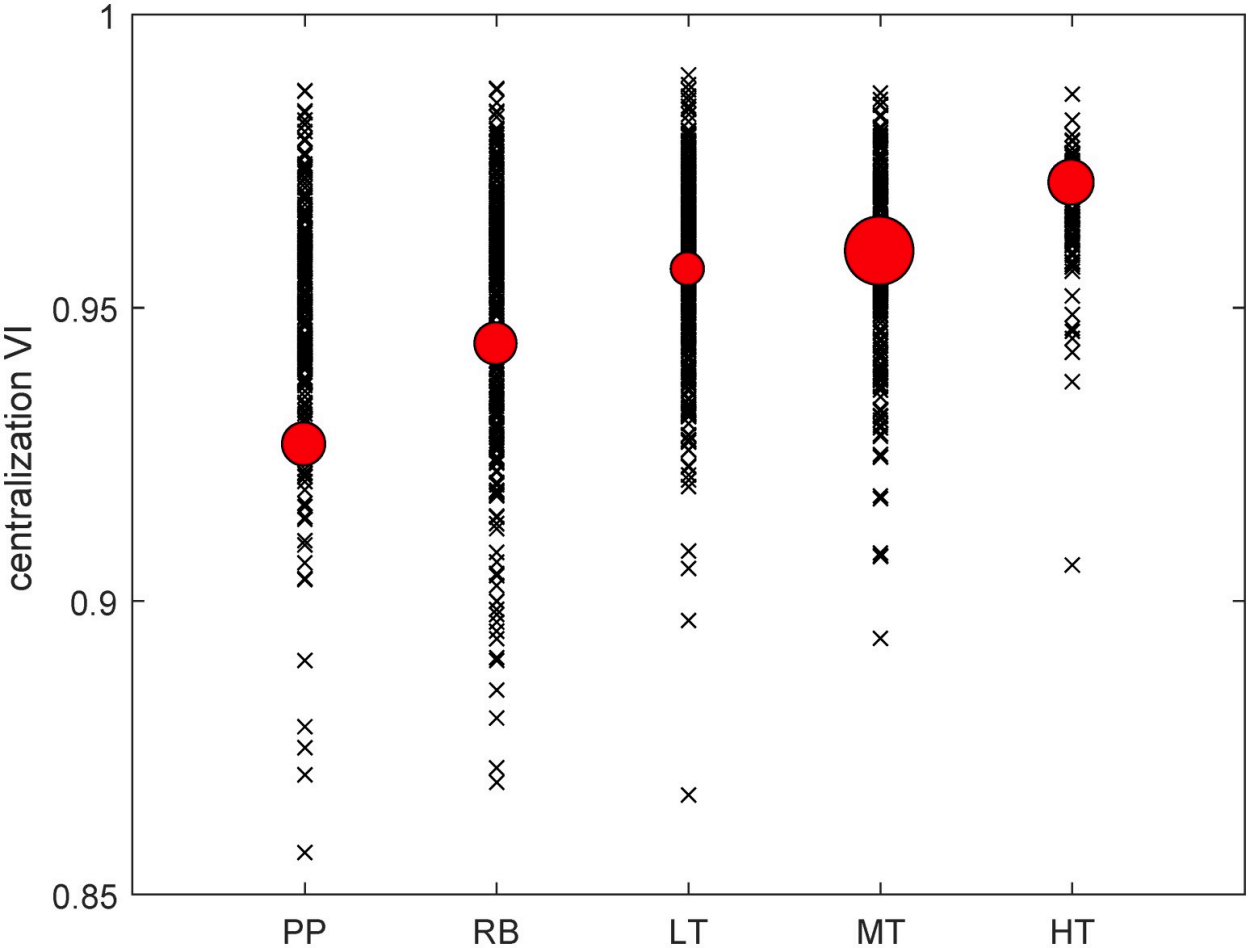
Complexity vs centralization in export data. The scatter plot reports the VI centralization values, for the complete set of 1,242 products, as a function of the technological class defined in Ref. [38] (PP: primary product; RB: resource based manufacture; LT: low-technology manufacture; MT: medium-technology manufacture; HT: high-technology manufacture). For each technological class, the position of the red dot corresponds to the mean (weighted by export value) of the centralization values and its size is proportional to the total export value of the class.

Fig 3 shows that, on the total set of products, complexity and centralization are indeed positively correlated (the test of the statistical significance for the nonzero slope is passed even with extremely restrictive p-values). Notably, this is consistently true for all the 3 × 3 complexity/centralization pairs. We corroborate this evidence by an independent analysis where, in place of the above complexity indicators, we use a standardized product classification based on technology content [38], defined exogenously from our data and measures, and we observe the VI centralization indicator of products in each technological category. This analysis is necessarily qualitative, since products are divided in discrete labeled classes and, consequently, a quantitative correlation analysis would be meaningless. Nonetheless, we obtain a clear evidence of an increasing trend of network centralization for increasing technological level, as illustrated in Fig 4, confirming our hypothesis. We see that all products classified as belonging to the high-tech category display a very high centralization, while primary products or low-technology manufactures include products with a definitely lower centralization.

**Fig 5:**
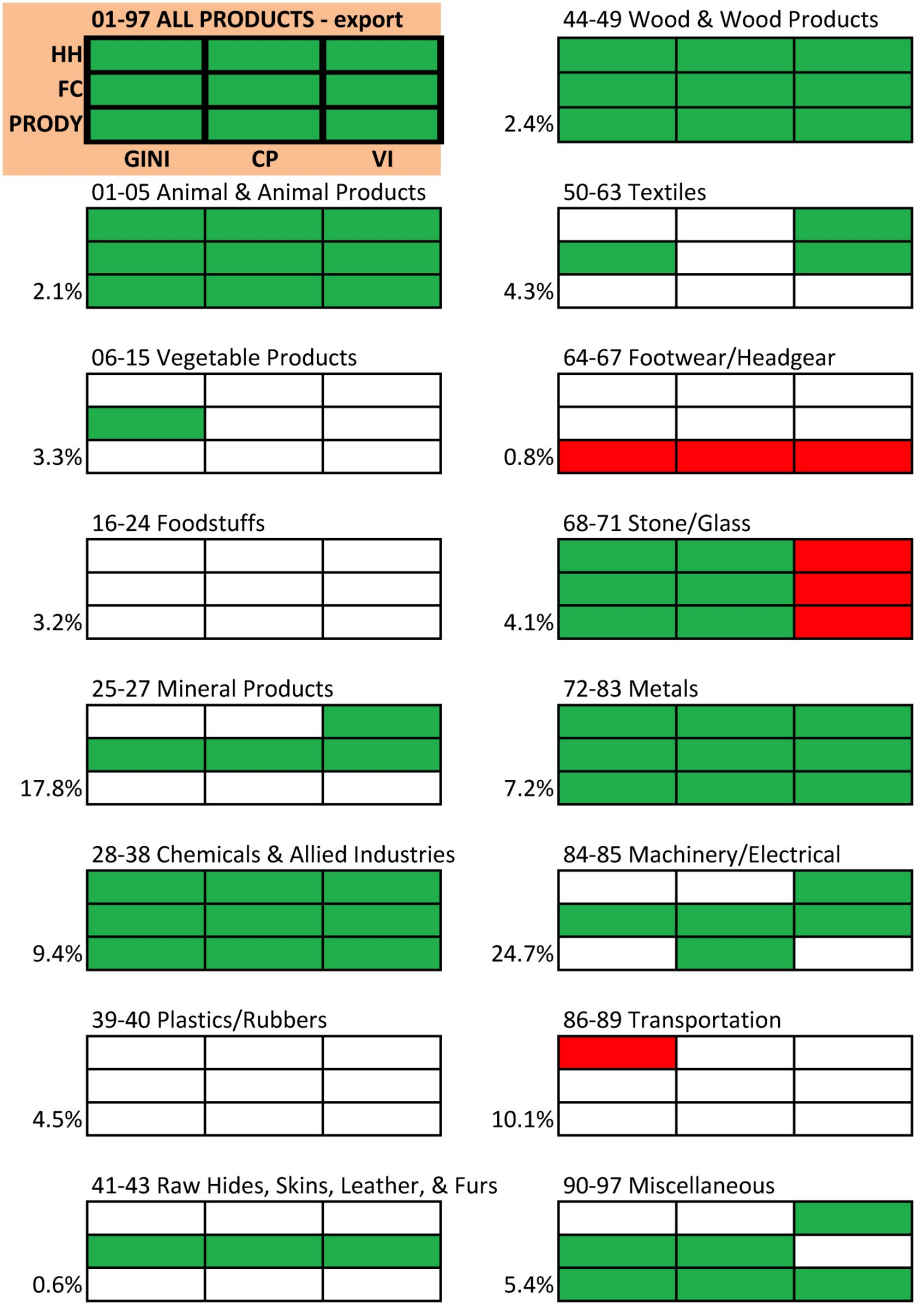
Complexity vs centralization in HS Sections export data: Consensus analysis. The top-left table refers to the complete set of 1,242 products (Fig 3), the other tables to the specified Section, whose share of the total world trade is on the bottom-left corner. Each table reports the results for the 3 × 3 combinations of complexity/centralization indicators (see top-left table for details). A green (resp., red) cell denotes a positive (resp., negative) correlation (p-value = 1%); a white cell denotes that correlation is not statistically significant.

To discover which categories of products are the main drivers of the above emerging pattern, we repeat the same analysis by partitioning the set of products into 15 sets based on the HS Classification by Section [39] (Fig 5). Taking into account the relative weight of each Section, i.e., the share of world trade, we have that the Sections most responsible of the overall complexity/centralization pattern are Machinery/Electrical, Chemicals, and Metals. Other Sections have the same consistent behavior (e.g., Animal & Animal Products) but a rather small trade share, whereas no Section evidences a clear opposite trend.

**Fig 6:**
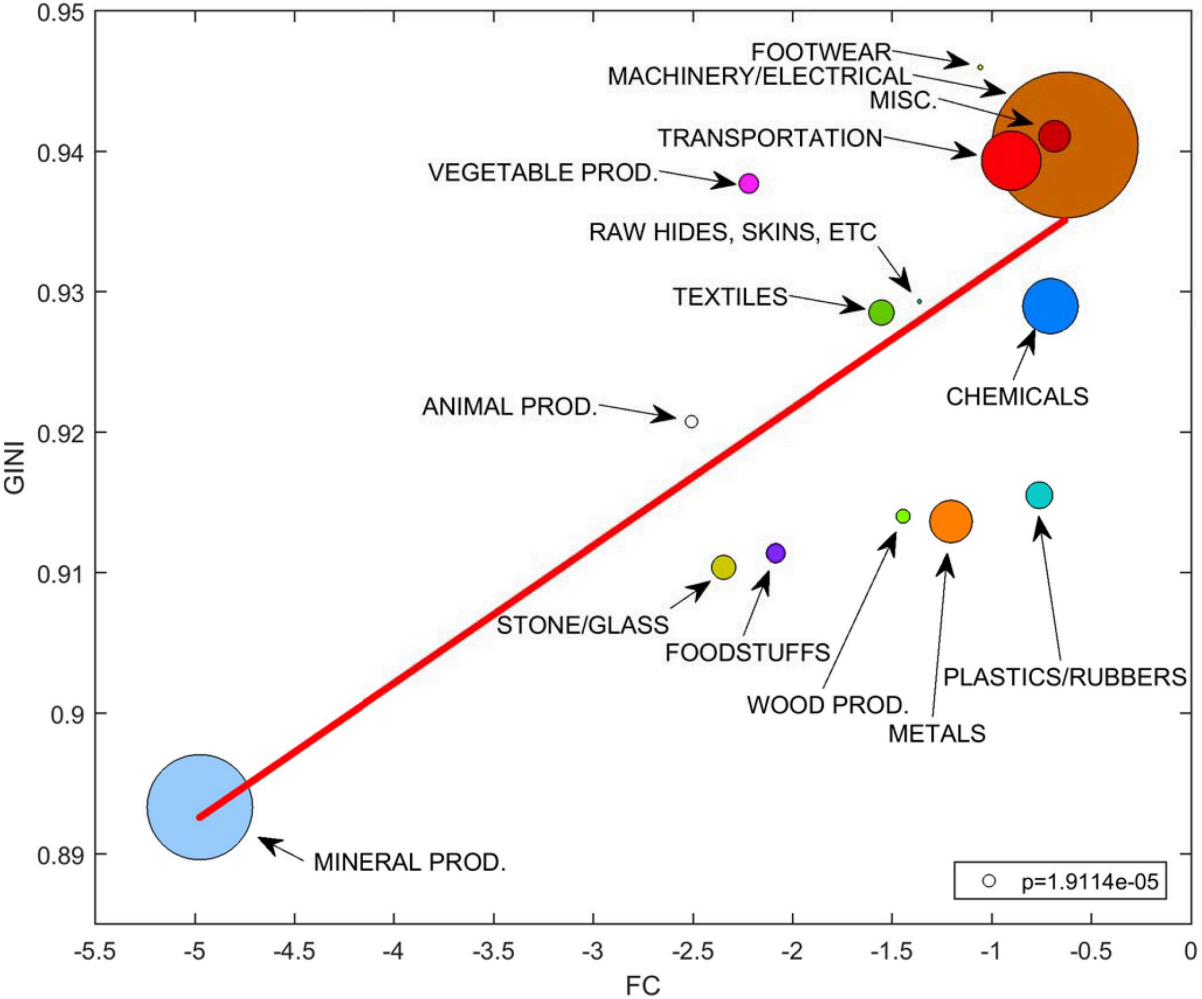
Complexity vs centralization in aggregate HS Sections. The scatter plot reports the complexity/centralization values (FC/GINI indices) for the fifteen Sections. The values are obtained as averages (weighted by trade volume) of the complexities/centralizations of the products of each Section. The marker size is proportional to the total trade volume of the Section. The weighted linear regression (red line) displays statistically significant positive slope (see p-value in the bottom-right corner).

Figs 3 and 5 show that, typically, products with larger complexity are distributed through a trade network with higher centralization, and that the same holds if we separately consider the most important (in terms of trade volume) subsets of products. The complementary analysis is instead to aggregate products by Section, and to compare the average complexity with the average centralization. Being more aggregate and containing products which are similar but might display different characteristics, the analysis by Sections could blur the association between complexity and centralization. Instead, the result (Fig 6) confirms that, even at this aggregate level, categories of products with larger complexities are associated to larger centralizations of their trade networks. On the bottom-left end of the plot in Fig 6, we observe mineral products characterized generally by low sophistication and complexity and displaying the lowest centralization measure among all observed categories. Machinery, electrical and transportation goods are instead in the upper-right corner of the plot. These are considered to be complex goods under all used definitions, as they are composed by a large number of inputs, they are highly differentiated and specialized products and they embody a high knowledge content. For these categories, the centralization is the highest observed. Therefore, also at this level of aggregation of products by Section, our hypothesis is significantly confirmed.

## Discussion

The results confirm the conjecture on the positive correlation between complexity of products and centralization of their trade networks. A complex product is obtained by combining different parts and inputs, produced applying specific knowledge and performing particular tasks. These procedures are not easily standardized and their knowledge content not easily transferable, with the possible exception of some limited parts. Therefore, these types of production take place in a small subset of locations and, consequently, complex goods can only be exported by a handful of countries, eventually yielding the observed centralization patterns.

Furthermore, many complex goods are produced through global value chains [40, 41], an organization of production where each phase takes place in a different country to benefit from specific inputs provided more efficiently. This production structure is typically organized around a hub coordinating the whole process. Hence, even if global value chains increase connectivity by generating many trade links between countries exchanging parts and inputs, the complex goods resulting from this organization are eventually exported by the final assembler, giving rise to a centralized structure of trade. The exceptions to the general correlation pattern refer to groups of products that might not meet the above characterization of complexity for the whole product class, but contain both simple, standardized types of goods, and very complex varieties (e.g., textiles or footwear). Other groups of products displaying a weak correlation are the ones that are not complex but tend to be produced in specific geographical areas (e.g., foodstuffs or wood products) because of the climate or geology of the region, and therefore still tend to have a centralized trade structure.

The high centralization observed for complex products drives the strong hierarchy of the overall trade network, given that they make up an important share of total trade. This implies that the pattern of trade links observed between countries is generally unevenly distributed and the possibility that shocks would average out at the world level is remote. Therefore, the current structure of the world trade network is indeed exposed to specific shocks: we emphasize that our centralization VI index explicitly quantifies the impact of shocks propagation from the central nodes. Considering such structure, it is not surprising that, in 2009, world trade experienced the strongest fall observed for over a century, after a serious economic crisis had hit some of the most central nodes—since then, trade flows have been much more volatile than in the previous decades [42], an undesirable feature that, given the persistence of such structure, could continue for long. While the literature highlights that uncertainty at the country level can have detrimental effects on local trade [43], fragility can play a similar effect on trade at the global level.

## Supporting information

S1 File Additional information and statistics on complexity and centralization indexes. (PDF)

## References

[1] International Monetary Fund. World Economic Outlook, https://www.imf.org/en/Publications/WEO/Issues/2016/12/31/Subdued-Demand-Symptoms-and-Remedies; 2016.

[2] Besedes T, Murshid AP. The effects of airspace closures on trade in the aftermath of Eyjafjallajökull, http://www.prism.gatech.edu/~tbesedes3/besedes-volcano.pdf; 2014.

[3] Carvalho VM, Nirei M, Saito YU, Tahbaz-Salehi A. Supply chain disruptions: Evidence from the Great East Japan Earthquake. Columbia Business School Research Paper. 2016;(17-5).

[4] De Benedictis L, Tajoli L. The world trade network. World Econ. 2011;34(8):1417–1454. 10.1111/j.1467-9701.2011.01360.x

[5] De Benedictis L, Nenci S, Santoni G, Tajoli L, Vicarelli C. Network Analysis of World Trade using the BACI-CEPII Dataset. Global Econ J. 2014;14(3–4):287–343.

[6] Acemoglu D, Carvalho VM, Ozdaglar A, Tahbaz-Salehi A. The network origins of aggregate fluctuations. Econometrica. 2012;80(5):1977–2016. 10.3982/ECTA9623

[7] Imbs J. Trade, finance, specialization, and synchronization. Rev Econ Stat. 2004;86(3):723–734. 10.1162/0034653041811707

[8] Schweitzer F, Fagiolo G, Sornette D, Vega-Redondo F, Vespignani A, White DR. Economic networks: The new challenges. Science. 2009;325(5939):422–425. 10.1126/science.1173644 19628858 10.1126/science.1173644

[9] Vespignani A. Complex networks: The fragility of interdependency. Nature. 2010;464(7291):984–985. 10.1038/464984a 20393545 10.1038/464984a

[10] Albert R, Jeong H, Barabasi A. Error and attack tolerance of complex networks. Nature. 2000;406(6794):378–382. 10.1038/35019019 10935628 10.1038/35019019

[11] Cohen R, Erez K, ben Avraham D, Havlin S. Breakdown of the internet under intentional attack. Phys Rev Lett. 2001;86(16):3682–3685. 10.1103/PhysRevLett.86.3682 11328053 10.1103/PhysRevLett.86.3682

[12] Barrat A, Barthélemy M, Vespignani A. Dynamical Processes on Complex Networks. Cambridge University Press; 2008.

[13] Barabási AL. Network Science. Cambridge University Press; 2016.

[14] Carvalho VM. From micro to macro via production networks. J Econ Perspect. 2014;28(4):23–48. 10.1257/jep.28.4.23

[15] Contreras MGA, Fagiolo G. Propagation of economic shocks in input-output networks: A cross-country analysis. Phys Rev E. 2014;90(6):062812 10.1103/PhysRevE.90.062812 10.1103/PhysRevE.90.062812 25615153

[16] Korniyenko Y, Pinat M, Dew B. Assessing the fragility of global trade: The impact of localized supply shocks using network analysis. International Monetary Fund. 2017;(WP 17/30).

[17] Gabaix X. The granular origins of aggregate fluctuations. Econometrica 2011;79(3):733–772. 10.3982/ECTA8769

[18] CEPII-BACI database. http://www.cepii.fr/CEPII/en/bdd_modele/presentation.asp?id=1; accessed April 2017.

[19] Rauch JE. Business and social networks in international trade. J Econ Lit. 2001;39(4):1177–1203. 10.1257/jel.39.4.1177

[20] Costinot A. On the origins of comparative advantage. J Int Econ. 2009;77(2):255–264. 10.1016/j.jinteco.2009.01.007

[21] Hidalgo CA, Hausmann R. The building blocks of economic complexity. Proc Natl Acad Sci USA. 2009;106(26):10570–10575. 10.1073/pnas.0900943106 19549871 10.1073/pnas.0900943106 PMC2705545

[22] Hausmann R, Hidalgo CA, Bustos S, Coscia M, Simoes A, Yildirim MA. The Atlas of Economic Complexity: Mapping Paths to Prosperity. Cambridge, MA: MIT Press; 2014.

[23] Caldarelli G, Cristelli M, Gabrielli A, Pietronero L, Scala A, Tacchella A. A network analysis of countries’ export flows: Firm grounds for the building blocks of the economy. PLoS One. 2012;7(10):e47278 10.1371/journal.pone.0047278 23094044 10.1371/journal.pone.0047278 PMC3477170

[24] Tacchella A, Cristelli M, Caldarelli G, Gabrielli A, Pietronero L. A new metrics for countries’ fitness and products’ complexity. Sci Rep. 2012;2:723 10.1038/srep00723 23056915 10.1038/srep00723 PMC3467565

[25] Cristelli M, Gabrielli A, Tacchella A, Caldarelli G, Pietronero L. Measuring the intangibles: A metrics for the economic complexity of countries and products. PLoS One. 2013;8(8):e70726 10.1371/journal.pone.0070726 23940633 10.1371/journal.pone.0070726 PMC3733723

[26] Mealy P, Doyne Farmer J, Teytelboym A. Interpreting economic complexity. arXiv:1711.08245 2018 10.1126/sciadv.aau1705 PMC6326748 30662945

[27] Wu R-J, Shi G-Y, Zhang Y-C, Mariani MS. The mathematics of non-linear metrics for nested networks. Phys A. 2016;460:254–269. 10.1016/j.physa.2016.05.023

[28] Pugliese E, Zaccaria A, Pietronero L. On the convergence of the fitness-complexity algorithm. Eur Phys J Special Topics 2016;225:1893–1911. 10.1140/epjst/e2015-50118-1

[29] Hausmann R, Hwang J, Rodrik D. What you export matters. J Econ Growth. 2007;12(1):1–25. 10.1007/s10887-006-9009-4

[30] Newman MEJ. Networks: An Introduction. Oxford University Press; 2010.

[31] Bustos S, Gomez C, Hausmann R, Hidalgo CA. The dynamics of nestedness predicts the evolution of industrial ecosystems. PLoS One 2012;7:e49393 10.1371/journal.pone.0049393 23185326 10.1371/journal.pone.0049393 PMC3501524

[32] Ermann L, Shepelyansky D. Ecological analysis of world trade. Phys Lett A 2013;377:250 10.1016/j.physleta.2012.10.056

[33] Alves LGA, Mangioni G, Cingolani I, Aparecido Rodrigues F, Panzarasa P, Moreno Y. The nested structural organization of the worldwide trade multi-layer network. arXiv:1803.02872 2018. 10.1038/s41598-019-39340-w PMC6393514 30814565

[34] Della Rossa F, Dercole F, Piccardi C. Profiling core-periphery network structure by random walkers. Sci Rep. 2013;3:1467 10.1038/srep01467 23507984 10.1038/srep01467 PMC3601366

[35] Piccardi C. Finding and testing network communities by lumped Markov chains. PLoS One. 2011;6(11):e27028 10.1371/journal.pone.0027028 22073245 10.1371/journal.pone.0027028 PMC3207820

[36] Dall’Asta L, Barrat A, Barthelemy M, Vespignani A. Vulnerability of weighted networks. J Stat Mech-Theory Exp. 2006; p. P04006.

[37] Ma Y, Qu B, Zhang Y. Complex goods’ exports and institutions: Empirics at the firm level. Rev Int Econ. 2012;20(4):841–853. 10.1111/j.1467-9396.2012.01059.x

[38] Lall S. The technological structure and performance of developing country manufactured exports, 1985-98. Oxf Dev Stud. 2000;28:337–369. 10.1080/713688318

[39] UN Trade Statistics. HS 2002 Classification by Section, https://unstats.un.org/unsd/tradekb/Knowledgebase/50043/HS-Classification-by-Section; accessed March 2018.

[40] Baldwin R. In: Feenstra RC, Taylor AM, editors. Globalization in an Age of Crisis: Multilateral Economic Cooperation in the Twenty-First Century. Chicago, IL: University of Chicago Press; 2013.

[41] Elms D, Low P. Global Value Chains in a Changing World. WTO, Fung Global Institute, Temasek Foundation; 2013.

[42] Bennett FR, Lederman D, Pienknagura SJ, Rojas D. The volatility of international trade flows in the 21st century: whose fault is it anyway? The World Bank; 2016. WPS 7781.

[43] Anderson J, Marcouiller D. Insecurity and the pattern of trade: An empirical investigation. Rev Econ Stat. 2002;84(2):342–352. 10.1162/003465302317411587

