# Supplementary material for: Complexity, centralization, and fragility in economic networks
Source: PLoS One. 2018 Nov 29;13(11):e0208265. doi: 10.1371/journal.pone.0208265 (PMC6264856; doi:10.1371/journal.pone.0208265)
Supplement: S1 File — (PDF) [file pone.0208265.s001.pdf]

Supporting information: Complexity, centralization, and fragility in economic networks

Carlo Piccardi[1,*] **and Lucia Tajoli**[2]

[1]Department of Electronics, Information, and Bioengineering, Politecnico di Milano, Piazza Leonardo da Vinci 32, 20133 Milano, Italy
[2]Department of Management, Economics, and Industrial Engineering, Politecnico di Milano, Via Lambruschini 4B, 20156 Milano, Italy
[*]carlo.piccardi@polimi.it

Complexity indexes

Figure A shows the relationship among the three indexes of complexity defined in the main text. As expected, they are overall strongly (positively) correlated. However, the deviation from the line of best linear interpolation is quite large and many outliers are observed. This suggests considering the entire pool of indicators in order to have robust results.

Centralization indexes

For each product, we analyze the largest weakly connected component (LWCC) of the trade network, so preserving directionality and weights but removing isolated nodes (i.e., countries not participating in the trade of the product) or small isolated subnetworks. The size of the LWCC, over the 1,242 products, ranges from 2 to 220, with mean 175.95 (median 189). Figure B points out that very small networks are associated to products with negligible total trade. Consequently, even if the values of whatever centralization index are scarcely reliable (if ever well defined) on such small networks, the role they have in the regression analysis (see main text) is negligible, given that regressions are weighted with total product trades.

Figure C shows the relationship among the three indicators of centralization defined in the main text. Despite they are the result of very different approaches, they are strongly (positively) correlated. Important discrepancies exist for very small networks (omitted in the figure) which, however, have negligible impact on the main results (see main text) because the associated trade volumes are negligible too, as pointed out in Fig. B.

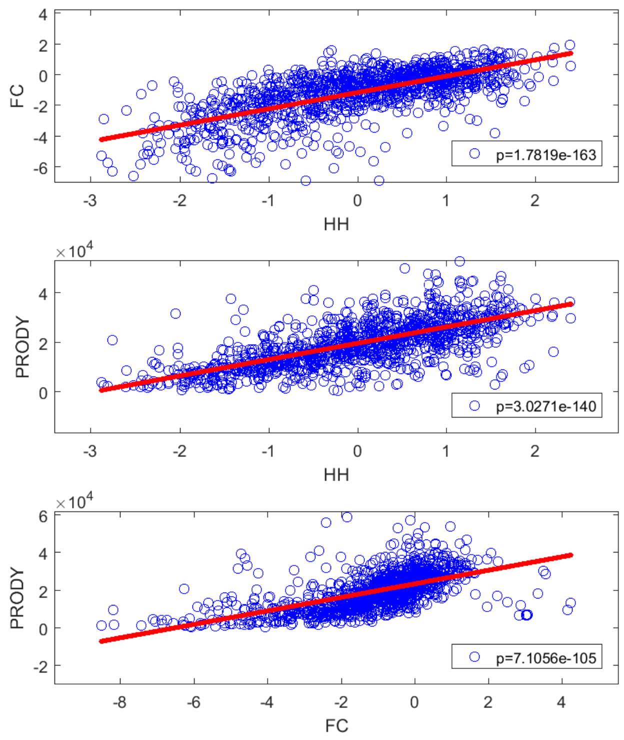

Figure A. Comparing complexity indexes. The scatter plots display pairwise comparisons of the three complexity indexes HH, FC, and PRODY, for the complete set of 1,242 products.

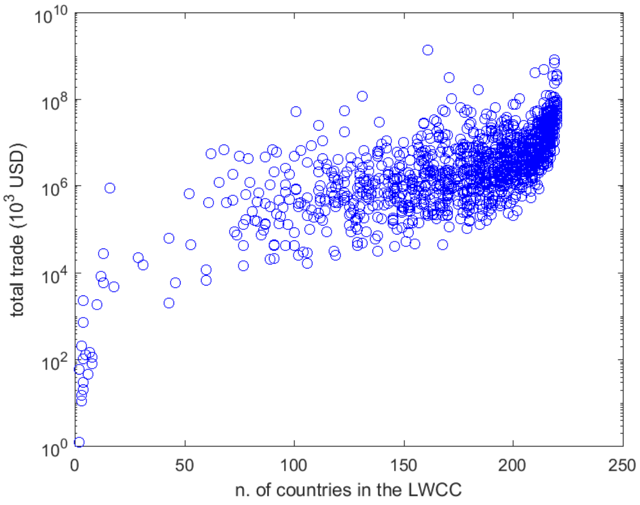

Figure B. Size and trade volumes of the largest weakly connected components (LWCCs). The scatter plot displays, for each product, the size (number of countries) of the LWCC and the total world trade volume. Very small networks are associated to negligible trade volumes.

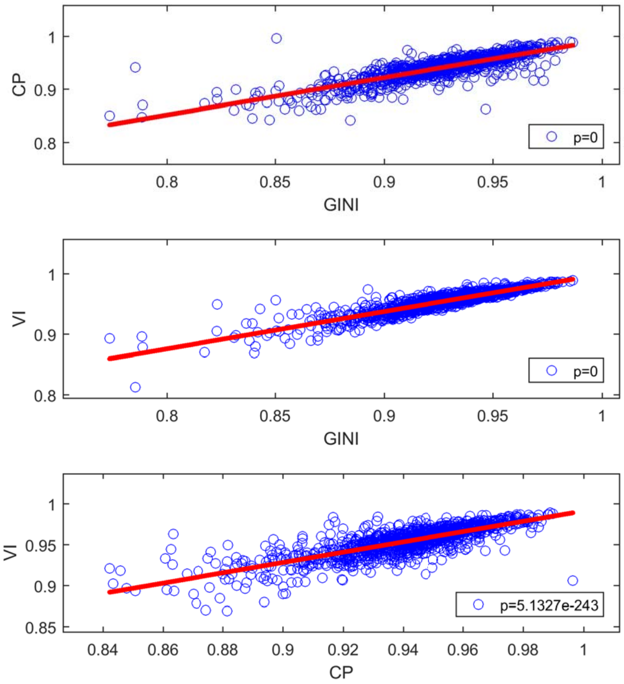

Figure C. **Comparing centralization indexes.** The scatter plots display pairwise comparisons of the three centralization indexes GINI, CP, and VI, for the complete set of products (trade networks with less than 20 nodes have been omitted from the plots).
